# Ability of Wearable Accelerometers-Based Measures to Assess the Stability of Working Postures

**DOI:** 10.3390/ijerph19084695

**Published:** 2022-04-13

**Authors:** Liangjie Guo, Junhui Kou, Mingyu Wu

**Affiliations:** Department of Safety Engineering, Faculty of Engineering, China University of Geosciences, Wuhan 430074, China; cugkou@cug.edu.cn (J.K.); 20151001279@cug.edu.cn (M.W.)

**Keywords:** working postural stability, balance assessment, wearable accelerometers, inertial measurement units, machine learning

## Abstract

With the rapid development and widespread application of wearable inertial sensors in the field of human motion capture, the low-cost and non-invasive accelerometer (ACC) based measures have been widely used for working postural stability assessment. This study systematically investigated the abilities of ACC-based measures to assess the stability of working postures in terms of the ability to detect the effects of work-related factors and the ability to classify stable and unstable working postures. Thirty young males participated in this study and performed twenty-four load-holding tasks (six working postures × two standing surfaces × two holding loads), and forty-three ACC-based measures were derived from the ACC data obtained by using a 17 inertial sensors-based motion capture system. ANOVAs, *t*-tests and machine learning (ML) methods were adopted to study the factors’ effects detection ability and the postural stability classification ability. The results show that almost all forty-three ACC-based measures could (*p* < 0.05) detect the main effects of Working Posture and Load Carriage, and their interaction effects. However, most of them failed in (*p* ≥ 0.05) detecting Standing Surface’s main or interaction effects. Five measures could detect both main and interaction effects of all the three factors, which are recommended for working postural stability assessment. The performance in postural stability classification based on ML was also good, and the feature set exerted a greater influence on the classification accuracy than sensor configuration (i.e., sensor placement locations). The results show that the pelvis and lower legs are recommended locations overall, in which the pelvis is the first choice. The findings of this study have proved that wearable ACC-based measures could assess the stability of working postures, including the work-related factors’ effects detection ability and stable-unstable working postures classification ability. However, researchers should pay more attention to the measure selection, sensors placement, feature selection and extraction in practical applications.

## 1. Introduction

Maintaining body balance is essential for humans in performing daily activities, and people with postural instability would be confronted with difficulty in completing desired actions [1,2]. Postural instability is one of most common factors related to injuries from falling [3], especially for the people who are likely to be exposed to a high risk of falls because of their poor health condition or dangerous working environment, such as Parkinson’s or stroke disease patients, obese people, older adults, construction workers, etc. [4,5,6]. For workers, e.g., construction workers, occupational falls are a serious problem, which leads to substantial injury and economic consequences [7]; it is reported worldwide that physical injuries caused by falls or related accidents are the leading cause of fatal injuries [8]. According to the American Bureau of Labor Statistics (2006 a & b), 32% of work-related deaths and 34% of nonfatal injuries in the construction industry were due to falls. Maintaining non-erect working postures while completing working tasks requires dynamic or static postural stresses [9]. Not only maintaining the static working postures, but also performing volitional movements, such as those needed transition from one posture to another, necessitate movement patterns that challenge the stable state of balance control and increase vulnerability to loss of balance and the risk of falling [10]. Therefore, assessing the stability of working postures is necessary and important for damage reduction and occupational fall prevention.

The measures to assess the stability of working postures can be classified into two groups: subjective and objective measures [11]. Perception of postural stability rated by using a visual scale is a widely used as the subjective measure, and it has a good reliability and validity [12,13,14]. However, the subjective measures are mainly based on the judgment of the person, and are easily influenced by the observer’s personality or experience, which is subject to cognitive bias [15]. In contrast, the objective evaluation method qualifies the subject’s body’s stability level by using the equipment, which is more impartial compared with the subjective evaluation methods. Center of pressure (COP) trajectory- based measures are the traditional and most widely-used objective measures [16,17,18,19,20], which are sometimes used as the gold standard. However, researchers have to rely on force plates (e.g., AMTI Force Plate, AMTI, Watertown, MA, USA) to obtain the COP data, which is quite expensive, difficult to set up and cumbersome to transport [21]. Recently, inertial sensor technology has been widely used in the balance- related research in the fields of biomechanics [22,23,24,25], ergonomics and human factors [26,27], sports science [28,29,30,31,32,33,34] and virtual or augmented reality [35,36,37], because the miniature inertial sensors are cost effective, wearable, compact and lightweight. With the rapid development and widespread application of wearable inertial sensors in the above fields, it is increasingly being used in the assessment of human body balance or postural stability [38,39,40,41,42,43].

Many kinds of ACC-based measures have been studied, such as Root-Mean-Square (RMS) [44,45,46,47], range [48,49,50], mean frequency [48,51], sway velocity [52,53,54], sway area [48,55], and mean distance [48,51]. By using the above ACC-based measures, previous studies have focused more on the effects of some fall-risk related factors, like age [52], health condition [44], working atmosphere [56], or the parameters referred to above to distinguish different sample groups, like young and old people [57,58], both healthy and those that are patients (usually patients with Parkinson’s, concussion, and stroke, etc.) [59,60]. Some researchers have reviewed the present literature to summarize the application status of inertial sensors in human balance assessment. For example, Ghislieri et al. summarized the state-of-the-art literature that focused on evaluating human standing balance using inertial sensors, they highlighted the main applications in active aging and clinics, and discussed the best data acquisition protocols [61]. Gordt et al. reviewed current studies on the effectiveness of wearable sensors-based training for improving of the functional performance, and they found positive effects of this kind of training [62]. Hasegawa et al. studied the most sensitive inertial sensors-based measures of balance dysfunction that differ between Parkinson’s patients and healthy people, and they found four measures that could be used for clinical trials focused on improving balance dysfunction [59]. Hubble et al. examined the current research that estimated standing and walking balance in Parkinson’s patients utilizing wearable inertial sensors, and they summarized the best sensor types, locations and outcomes [63]. However, limited studies were found that systematically investigated the ability of ACC-based measures to detect the effects of work-related factors and the ability to classify stable and unstable working postures. Considering the portable and wearable inertial sensor technology has significant advantages in human motion capture and balance assessment, especially for the application in the working environment. Moreover, to assess the stability of working postures it is necessary and important for damage reduction and occupational falls prevention, and has sparked intense interest among researchers. For this reason, this study aims to systematically investigate the abilities of ACC-based measures to assess the stability of working postures in terms of the ability to detect the effects of work-related factors and the ability to classify stable and unstable working postures. The findings could provide some reference for researchers to select ACC-based measures, choose optimal sensor placement locations, and determine best test protocols when using the ACC-based measures for working postural stability assessment.

## 2. Methods

### 2.1. Participants

Thirty young males (age 28.20 ± 4.66 years; height 171.62 ± 6.62 cm; weight 68.09 ± 6.89 kg) without neurological diseases or self-reported musculoskeletal issues (i.e., without any body balance-related issues) volunteered to participate in this study. Each participant signed informed consent on this experiment protocol, which was improved by the Institutional Review Board of Korea’s Advanced Institute of Science and Technology (KAIST, Approval Number KH2017-38).

### 2.2. Experimental Design

#### 2.2.1. Independent Variables

The experiment was a full factorial within-subject repeated measures design. Three independent variables were considered: (1) Working Posture (WP, six levels): natural standing upright (P1), standing upright with arms raised to nineteen degrees in the sagittal plane (P2), squatting (P3), bending forward (P4), bending forward with left-turning (P5), and overhead carrying (P6); (2) Surface Slope (SS, two levels): flat and inclined (18 degree slope) standing surfaces; (3) Load Carriage (LC, two levels) in hands without (0 kg) and with (10 kg) load carriage in hands. The above three independent variables were designed concerning the real working scenarios of construction workers and masons. For example, the six different working postures selected in this study are frequently used when a mason lays stones, cinder blocks, or bricks in the construction of building walls.

#### 2.2.2. Dependent Variables

The dependent variable was postural stability (PS, i.e., stability level of different working postures) under different experimental conditions. The postural stability was measured using both subjective and objective measures in this study with references to previous studies [64,65]. The subjective measure used in this study was the perception of postural stability (PPS), which reflects the participant’s subjective perception of difficulty in keeping their balance. This measure (i.e., PPS) has proven to be reliable and valid in assessing postural stability by many previous studies [12,13,14].

**Figure 1:**
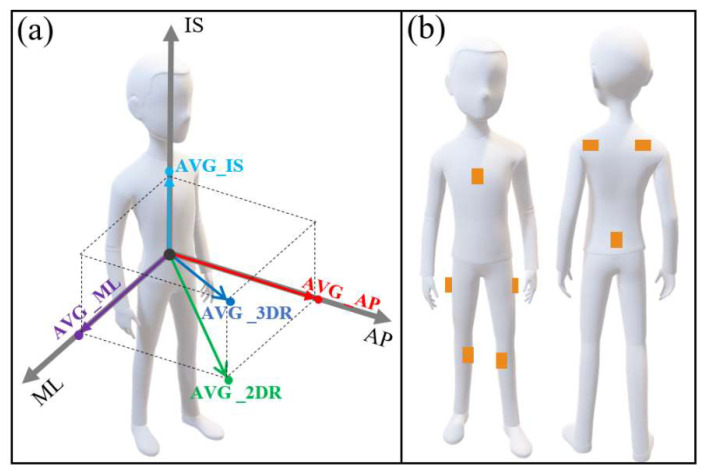
(**a**) Diagram of the ACC-based measures in different directions (such as ACC-based Average (AVG), for example); (**b**) Configuration of the eight IMU sensors used in this study.

Two kinds of objective measures utilized in this research were center of pressure- (COP) based and acceleration- (ACC) based measures. (a) COP-based measures: Only one COP-based measure, mean velocity of COP in the anterior-posterior direction (COPV_AP) [20], was derived from COP data. COPV_AP and PPS were considered as the objective and subjective golden references of the postural stability, respectively. Since both of them have proven to be reliable and valid, and our previous studies had shown these two measures could provide adequate discriminating power for detecting the effects, including main and interaction effects, of the investigated factors [11,13]. (b) ACC-based measures: Considering that the objective of this study is to evaluate the ability of ACC-based measures in assessing the stability of working postures, forty-three ACC-based measures (see Table 1 and Figure 1a) derived from the acceleration data, were calculated. Both COP-based and ACC-based measures were calculated with references to previous studies [18,19,20,47,48,52,54,60,66,67], and the calculation methods of some parameters described by Prieto et al. [20] and Raymakers et al. [67], which were applied to the COP signals, were applied to the acceleration signals in this research [68].

#### 2.2.3. Experimental Apparatus

An 11-point (0–10) Likert difficulty rating scale was used to assess the participant’s perception of the difficulty (0 = not at all; 5 = moderately; 10 = extremely) in keeping his balance. A Wii Balance Board (Nintendo, Kyoto, Japan) interfaced to a PC using a custom-written software was used for center of pressure data recording, at a frequency of 40 HZ. The inertial sensor-based motion capture system, Xsens MVN Link (Xsens Technologies B.V., Enschede, The Netherlands), powered by its matching software Xsens MVN Analyze was used for acceleration data recording at a frequency of 240 HZ. This system was composed of 17 IMU sensors which were placed over the full body (17 different body segments) [69]. Furthermore, a custom-made, 18 degree sloped wooden platform was used as the inclined standing surface. A 30 cm × 21 cm × 21 cm plastic foam box, empty (~0 kg) or fully filled with paper (10 kg), was used as the load.

#### 2.2.4. Experimental Tasks

**Figure 2:**
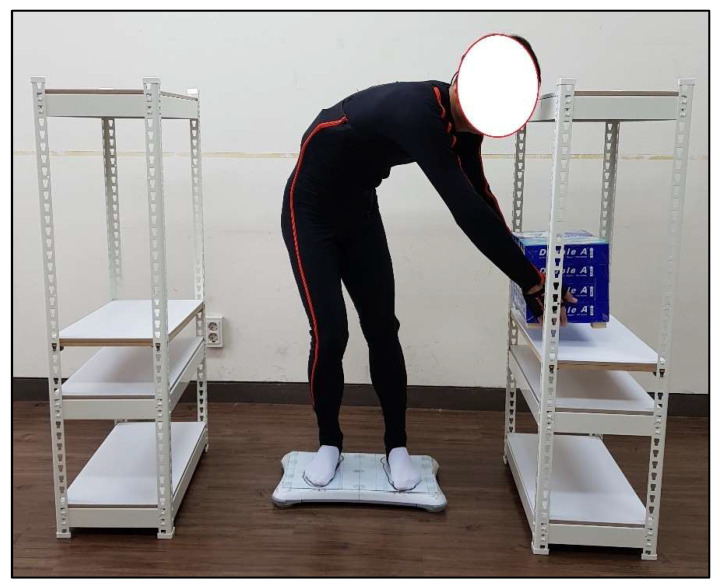
Schematic representation of the experimental tasks (take flat standing surface × 10 kg load carriage × Posture 5 as an example).

Each participants was instructed to wear the Xsens MVN Link system by following the official instructions from Xsens, which were that 17 IMU sensors were attached on the pelvis, sternum (eighth thoracic vertebra, T8), back of the head, shoulders, upper arms, forearms, hands, upper legs, lower legs and feet [69]. All the participants were tested without wearing shoes, and non-slip socks were provided by the tester to the eliminate potential effects of different shoes on postural stability. Figure 2 shows the schematic representation of the experimental tasks (take flat standing surface × 10 kg load carriage × Posture 5 as an example). Next, each of the participants was instructed to stand naturally on the standing surface (flat & inclined) with feet in a comfortable position and to hold the foam box (~0 kg & 10 kg) with both hands for ten seconds using six working postures (P1–P6). Twenty-four load-holding tasks (six working postures × two standing surfaces × two holding loads) in total were performed by each participant, and each task was repeated twice. During each task, the COP and acceleration data were collected. Right after finishing each task, the participant was asked to rate his perception of the difficulty level to keep his balance during the load-holding task. The experimental tasks were performed in random order. In addition, two research assistants were arranged to stand beside the participants to protect them from falling or other accidents.

### 2.3. Data Analysis

The abilities of ACC-based measures to assess the stability of working postures were investigated from two dimensions: the ability to detect the effects of work-related factors and the ability to classify stable and unstable working postures using a machine learning method. The PPS and COPV-AP were used as the golden references of the postural stability.

#### 2.3.1. Data Preprocessing

For body stability assessment, only the acceleration data derived from eight sensors were extracted, and these were attached to the following eight segments (see Figure 1b): pelvis, sternum (T8), shoulders, upper legs, and lower legs. The raw two-dimensional COP and three-dimensional acceleration data were filtered using a fourth order zero-phase low pass Butterworth filter. The filtered data were used for calculating the above-mentioned COP-based and ACC-based measures.

#### 2.3.2. Factors’ Effects Detection Based on Statistical Analysis

In order to study the ability of each ACC-based measure to detect the effects of work-related factors (i.e., WP, SS, and LC in this study) on postural stability, three-way repeated-measures ANOVAs and paired sample *t*-tests were performed based on each measure. The normality test was conducted using the Shapiro-Wilk test. The spherical assumption was checked using Mauchly’s test of sphericity, and the Greenhouse-Gasser correction was applied when this assumption was violated [70]. The statistical analysis was conducted using the SPSS statistical package (v. 26.0, IBM Corp., Armonk, NY, USA), and the postural stability measures calculations were carried out in MATLAB (v. R2020a, Math Works Inc., Natick, MA, USA).

#### 2.3.3. Postural Stability Classification Based on Machine Learning 

The ability of ACC-based measures to classify stable and unstable working postures was studied using machine learning methods.

(1) Feature selection and extraction. The following twelve features were selected and extracted based on the raw acceleration data: mean, range, variance, standard deviation, root mean squared, skewness, kurtosis, and the first five fast Fourier transformation (FFT) coefficients. In order to identify and avoid overfitting, and evaluate the performance of using different combinations of sensors and/or features, the twelve features and eight sensors were split into three feature sets and three sensor combinations, as shown in Table 2. After feature extraction, z-score standardization was applied to transform features to comparable scales.

(2) Classifier selection. The following classifiers were used for models training, k-Nearest Neighbor (KNN), Gaussian Naive Bayes (GNB), Kernel Naive Bayes (KNB), Logistic Regression (LR), Discriminant Analysis (DA), Support Vector Machine (SVM), Decision Tree (DT), Bagged Trees (BT) and Optimizable Ensemble (OE) classifiers, which were provided in MATLAB Classification Learner toolbox [71]. The machine learning- based postural stability classification, including all related data processing, was conducted using MATLAB (v. R2020a, Math Works Inc., Natick, MA, USA), especially the Classification Learner toolbox of MATLAB, which was used for model training and testing.

(3) Classification performance. K-fold cross validation (*k* = 5 in this study) was performed to evaluate the machine learning models and that the dataset was divided into *k* subsets, one of which was kept as the validation set while the other *k* − 1 subsets were used as the training set. Such a process was repeated for each subset (i.e., *k* times in total), and then the average error across all trials was calculated, which was used for computing the classification accuracy according to the following equation [72,73].

(1) Accuracy=(TP+TN)/(P+N)∗100%

where *TP* and *TN* are true positive and true negative, respectively; *P* and *N* are the counts of positive and negative, respectively.

(4) Data labeling. The raw data set was labeled as ‘stable’ or ‘unstable’ with reference to the PPS and COPV_AP values, which were considered as the subjective and objective gold standard references of postural stability, respectively, as shown in Table 3.

## 3. Results

### 3.1. Ability to Detect the Effects of Work-Related Factors

As mentioned above, the ability to detect the effects of work-related factors on postural stability was studied by conducting several ANOVAs and *t*-tests using each of forty-three different measures, which were calculated based on the ACC data derived from the accelerometers attached on eight different segments. The statistical results using the ACC data of accelerometers attached on the pelvis and T8 are shown in Table A1 in the Appendix A. In addition, the results based on the other six segments’ ACC data (shoulders, upper legs, and lower legs) are summarized in Table A2, Table A3 and Table A4 in the Appendix A.

The *t*-test and ANOVA results (Table A1, Table A2, Table A3 and Table A4) show that all the ACC-based measures could (*p* < 0.05) detect the main effects of working posture (WP); almost all the measures (except for part of mean velocity (MV) related measures) were able to (*p* < 0.05) detect the main effect of load carriage (LC), and most of the measures failed (*p* > 0.05) in detecting the main effect of surface slope (SS). In particular, twenty-seven measures were based on pelvis ACC data, nineteen measures were based on left lower leg data, and ten measures were based on right lower leg data that successfully detected the SS’s main effects; this quantity was much larger than the quantity (three to five) of the measures based on the other five segments’ data. In contrast, there was no big difference in the numbers (thirty-nine to forty-three) of ACC-based measures, which could detect the main effects of WP and/or LC among the eight different segments. For the interaction effects’ detection ability, the results show that all of the ACC-based measures (except for part of mean velocity (MV) related measures) successfully detected (*p* < 0.05) the interaction effect between WP and LC, but most of the measures failed (*p* > 0.05) in detecting the SS’s interaction effects with the other two factors (i.e., SS × LC, SS × WP, and SS × LC × WP interactions). Besides, five measures (RMS_AP based on pelvis ACC data, FD_CC, MF_AP, MF_2DR, and MF_3DR based on left lower leg data, as shown in Table A1, Table A2, Table A3 and Table A4) successfully detected both the main and interaction effects of all three work-related factors.

Table 4 shows the numbers of ACC-based measures which successfully detected the factors’ effects. All of the forty-three measures were capable (*p* < 0.05) of detecting the main effects of WP, almost all of them (≥39) were capable (*p* < 0.05) of detecting the main effects of WP and the interaction effects LC × WP, which indicated an excellent performance in detecting the above effects. Conversely, most of the measures failed (*p* > 0.05) in detecting SS’s main or interaction effects.

### 3.2. Ability to Classify Stable and Unstable Working Postures

The classification results, i.e., the overall average classification accuracy for different sensor configurations and different feature sets using different classifiers, are presented in Table 5 (data labeling based on COPV_AP) and Table 6 (data labeling based on PPS).

(1) Data labeling with reference to COPV_AP.

In this part, the data sets were labeled using the average COPV_AP values. As shown in Table 3, the experimental task (i.e., data set) was labeled as Stable if its COP_AP value was smaller than the median of all COP_AP values, otherwise, it would be labeled as Unstable. The results (Table 5) show that the classification accuracy range for different SCs and FSs using different classifiers, was between 69.0% and 91.6%. The maximum accuracy was 91.6% by using the OE classifier based on the SC1 + FS1 data set, and the minimum was 69.0% by using the KNN (City Block) classifier based on the SC1 + FS2 data set. For each SC and each classifier, there was no major difference (within 2.4%) in the classification accuracy between using FS1 and FS3. However, compared to using FS1 or FS3, the classification performance using FS2 was much poorer (3.6%–21.7% lower in classification accuracy for different classifiers). No significant difference was found in the average (within 3.8%) or maximum (within 4.6%) classification accuracy between different SCs for each FS. Four classifiers (OE, OBT, DA, and SVM-Cubic) performed a bit better (2.5%–7.2% higher in the average accuracy) than the others in classifying stable and unstable working postures.

(2) Data labeling with reference to PPS.

Similarly, the experimental task (i.e., data set) was labeled as Stable if the PPS value was smaller than 5.0, and was labeled as Unstable if the PPS value was greater than or equal to 5.0. As shown in Table 6, the classification accuracy range, for different SCs + FSs using different classifiers, was between 68.8% and 87.4%. The maximum and minimum accuracy were 87.4% using KNN (City Block) based on the SC1 + FS1 data set, and 68.8% by using the DT classifier based on the SC3 + FS2 data set, respectively. For each SC and each classifier, there was no major difference (within 2.8%) in the classification accuracy between using FS1 and FS3. However, compared to using FS1 or FS3, the classification performance using FS2 was poorer (2.8%–12.1% lower in classification accuracy for different classifiers). No significant difference was found in the average (within 1.6%) or maximum (within 2.1%) classification accuracy between different SCs for each FS.

(3) Classification performance based only on Pelvis ACC data.

To further investigate the classification performance by only using one sensor, we repeated the above machine learning procedure based only on pelvis ACC data. Table 7 shows the results of the classification accuracies for different feature sets using different classifiers based only on Pelvis acceleration data. The result shows that the maximum classification accuracy was 90.5% (FS1 + OE) and 84.1% (FS1 + OE) using different labeling criteria, which were a little lower (1.0% and 2.0% respectively) than using SC3 (pelvis + T8, as shown in Table 6).

## 4. Discussion

This study investigated the abilities of ACC-based measures to assess the stability of working postures in terms of the ability to detect the effects of work-related factors and the ability to classify stable and unstable working postures using a machine learning method. The results show a good performance of these ACC-based measures. 

### 4.1. Ability in Factor Effects Detection

As shown in Table 4, all, or almost all, of the forty-three measures were capable to detect the main effects of WP and LC, and their interaction effects, which indicated an excellent performance in detecting the above effects. Conversely, most of the measures failed in detecting SS’s main or interaction effects. Our previous studies [11,13] had shown that there were statistically significant (*p* < 0.05) main effects of all the three factors, and statistically significant (*p* < 0.05) three-way and two-way interaction effects among them, which were based on both PPS- and COP-related (e.g., COPV_AP) measures. However, the 10-kg load carriage had a larger main effect on the body’s stability than the 18-degree inclined standing surface, so that the increase (3.05 in average PPS, *p* < 0.05; and 13.84 mm/s in average COPV_AP, *p* < 0.05) in the body’s instability caused by the 10-kg load in hands was larger than that (0.47 in average PPS, *p* < 0.05; and 3.36 mm/s in average COPV_AP, *p* < 0.05) caused by the 18-degree inclined surface. The above might explain why many ACC-based measures failed in detecting the SS’s effects since its effect size was relatively smaller.

Among the forty-three measures, five of them (RMS_AP based on Pelvis ACC data, FD_CC, MF_AP, MF_2DR and MF_3DR based on left lower leg data, see Table A1 and Table A4) could detect both the main and the interaction effects of all the factors, which are recommended ACC-based measures for working postural stability assessment. This finding was partly in line with the previous studies [45,54,74,75,76]. The results show that mean frequency measures (MF_AP, MF_ML, MF_2DR or MF_3DR), especially MF measures based on pelvis, upper or lower legs data, had a better overall performance than the other ACC-based measures (except for the SS*LC interaction effect detection based on T8 and shoulders data; see Table A1, Table A2, Table A3 and Table A4). 

For the placement of the accelerometer sensors, the pelvis and lower legs are recommended locations overall. The pelvis is a particularly a prime location for sensor placement, because the numbers of the measures based on pelvis ACC data, which were able to detect the factors’ effects, were larger than those based on the other segments’ data (except for SS*LC effect detection). That is because the pelvis is close to the body’s center of mass, especially during standing postures, and its kinestate could accurately reflect the body sway. Therefore, the pelvis was adopted by many previous studies for IMU sensor attachment in postural stability assessment [77,78,79]. There was also good performance of ACC measures in effects detection if the sensors were attached on the lower legs, especially on the left lower leg. As Ghislieri et al. [61] found, among the reviewed 47 articles that focused on standing balance assessment using wearable inertial sensors, 38 articles (80.9%) and 12 articles (25.5%) placed the sensors on the lower back (pelvis or closed to pelvis) and lower legs (shank), respectively. The lower legs were chosen [66,80,81] because the ankle control strategy was one of the most important balance control strategies [43,78] which might result in relatively larger changes in lower leg movement during standing [82,83]. In addition, the performance using T8, shoulders, or upper legs data was not good. There was no big significant difference among the eight different sensor placements in terms of LC, WP, and LC*WP effect detection (see Table 4).

### 4.2. Ability in the Classification of Stable and Unstable Postures Based on Machine Learning

In this study, the subjective perception of postural stability (PPS) and objective mean velocity of COP in anterior-posterior direction (COPV_AP) were taken as the gold standard of postural stability. Both of them had proven to be reliable and valid in assessing postural stability by previous studies [12,14,17,64,65,84], and could provide adequate discriminating power for detecting the main and interaction effects of all the factors investigated in this study [11,13]. In addition, the Nintendo Wii Balance Board and not a force plate was taken as the COP signal recording apparatus due to its advantages of low cost, portability, and easy setup. More importantly, it was reliable and valid in recording COP trajectory, which has been demonstrated in many previous studies [85,86,87]. For all of the above reasons, it should be reasonable that the raw data set (i.e., each experimental task) was labeled as ‘stable’ or ‘unstable’ with reference to PPS and COPV_AP values (see Table 3). If the data set was labeled based on the COPV_AP criterion, results (Table 5 and Table 6) show that the classification accuracies were higher, on the whole, than those based on the PPS criterion. It’s probably because the stability classification using the machine learning method was based on objective data, i.e., the features were extracted from the objective ACC signals, which caused the classification results to be more consistent with the objective COPV_AP measure.

The maximum classification accuracy reached 91.6% and 87.4%, respectively, using different labeling criteria. This performance was acceptable, which suggested the potential of the acceleration data in postural stability classification using the machine learning method. Our results show that the feature sets exerted a great influence on the classification performance. For each SC and most classifiers, the classification performance using FS2 was much poorer (2.8–21.7% lower in accuracy than using FS1 or FS3). There was a slight difference (within 2.8%) in the accuracy between using FS1 and FS3, which could be ignored. And consequently, Feature Set 1 (FS1, mean, range, variance, standard deviation, root mean squared, skewness and kurtosis) with less computation cost and better classification performance could be an ideal choice for postural stability classification among the three feature sets. 

Different from the feature set, there was no major difference in the average and maximum classification accuracy between different SCs for each FS and most classifiers. It indicated that the impact of sensor configuration turned out to be small. In this case, the third sensor configuration in this study (i.e., SC 3, pelvis + T8), with the minimum number of sensors, could be recommended as a first choice when the number of sensors needs to be reduced. As mentioned above, to further investigate the classification performance of only using one sensor, we repeated the above machine learning procedure based only on pelvis ACC data. The result (Table 7) shows that the maximum classification accuracy was 90.5% and 84.1% using different labeling criteria, which were a little lower (1.0% and 2.0% respectively) than using SC3 (pelvis + T8). This suggested that removing T8 sensor data would result in an overall decrease in the classification accuracy, but the decrease was relatively small. This is reasonable because the kinestate of the pelvis sufficiently reflected the motions of the whole body and, as mentioned above, it was frequently adopted by studies for IMU sensor attachment in balance assessment [77,78,79]. Therefore, the features extracted from pelvis ACC data used for classification models training should be more representative than those extracted from the other segments’ data.

It is important to point out that the factors that could influence human body balance are numerous and can be broadly classified into intrinsic and extrinsic factors [88]. Intrinsic factors are related to the functions of physiological or psychological systems, including fear of falling, visual, vestibular, cognitive, and musculoskeletal systems [17,89,90,91,92]. Extrinsic factors are various, such as the lifting load [93], standing surface slope [78,94], foot wear [95], etc. Body posture has proven to be an important extrinsic factor which could influence the stability of the body [13,96,97]. Therefore, the body stability status is the comprehensive outcome of multiple factors, and future studies are expected to investigate the potential of ACC-based measures in detecting the effects of other factors. 

The limitations of the research need to be acknowledged. Firstly, only three work-related factors (WP, SS and LC) were considered in this study, and caution should be taken when applying the findings if more other factors are considered, since various factors could lead to the imbalance of the body. Secondly, a machine learning method was used for studying the ability to classify stable and unstable working postures, and this study did not focus on hyperparameter optimization even though applying hyperparameter optimization might improve performance. Thirdly, the experimental tasks were selected based on our field studies, which are simulations of the working tasks of construction workers. All of the experimental data were collected in a laboratory setting, not on a construction site.

## 5. Conclusions

This study investigated the abilities of ACC-based measures to assess the stability of working posture in terms of the ability to detect the effects of work-related factors and the ability to classify stable and unstable working postures. The research achieved the following findings. Firstly, this study has proved that ACC-based measures have the abilities to assess the stability of working postures, including the work-related factors’ effects of detection ability and stable-unstable working postures classification ability. Secondly, five measures (RMS_AP based on Pelvis ACC data, FD_CC, MF_AP, MF_2DR and MF_3DR based on left lower leg data) are recommended for working postural stability assessment, and the pelvis and lower legs are recommended locations for sensor placement overall, with the pelvis being the first choice. Thirdly, compared with SC (Sensor Configuration, i.e., sensor placement locations), FS (Feature Set) exerted a greater influence on the classification accuracy when the machine learning method was used. In addition, researchers should pay more attention to the measure selection, sensor placement, feature selection and extraction when using ACC-based measures for working on postural stability assessment.

## Figures and Tables

**Table 1:** Acceleration based postural stability measures adopted in this study.

Measures (Abbr.)	Explanations
Average (AVG) [66]	Average of ACC ^1^ in AP ^2^, ML ^3^, IS ^4^, 2DR ^5^, and 3DR ^6^ directions: AVG_AP, AVG_ML, AVG_IS, AVG_2DR, and AVG_3DR (m/s^2^).
Range (RNG) [48,54]	Range of ACC in AP, ML, IS, 2DR, and 3DR directions: RNG_AP, RNG_ML, RNG_IS, RNG_2DR, and RNG_3DR (m/s^2^).
Root mean squared (RMS) [47,48,52]	Root mean squared of ACC in AP, ML, IS, 2DR, and 3DR directions: RMS_AP, RMS_ML, RMS_IS, RMS_2DR, and RMS_3DR (m/s^2^).
Sway area (ARE) [48,54,60]	Area of sway (ARE_SW), area spanned from the ACC signals per unit of time (mm^2^/s^5^).
	Area of 95% confidence circle (ARE_CC) and 95% confidence ellipse (ARE_CE), that encapsulates the sway path derived from ACC per unit of time (mm^2^/s^5^).
Fractal dimension (FD) [20]	Fractal dimension based on 95% confidence circle (FD_CC) and 95% confidence ellipse (FD_CE).
Length (LEN) [48,54]	Total length of ACC trajectory in AP, ML, 2DR, and 3DR directions: LEN_AP, LEN_ML, LEN_2DR, and LEN_3DR (m/s^2^).
Mean distance (MD) [48,54]	Mean distance from center of ACC trajectory in AP, ML, 2DR, and 3DR directions: MD_AP, MD_ML, MD_2DR, and MD_3DR (m/s^2^).
Mean frequency (MF) [48,54]	Mean frequency of ACC power spectrum in AP, ML, 2DR, and 3DR directions: MF_AP, MF_ML, MF_2DR, and MF_3DR (HZ).
Mean velocity (MV) [48,54]	First integral of ACC signals in AP, ML, 2DR, and 3DR directions: MV_AP, MV_ML, MV_2DR, and MV_3DR (m/s).
Planar deviation (PD) [67]	Planar deviation in displacement (PD_P) and velocity (PD_V).
Phase plane parameter (PP) [67]	Phase plane parameter (square root of the sum of variances of displacement and velocity).
Root mean squared distance (RMSD) [20]	Root mean squared distance from center of ACC trajectory in AP, ML, 2DR, and 3DR directions: RMSD_AP, RMSD_ML, RMSD_2DR, and RMSD_3DR (m/s^2^).

^1^ ACC, acceleration; ^2^ AP, anterior-posterior; ^3^ ML, medial-lateral; ^4^ IS, inferior-superior (i.e., vertical); ^5^ 2DR, two-dimensional resultant (in transverse plane); ^6^ 3DR, three-dimensional resultant.

**Table 2:** Feature sets and sensor combinations for the training of the machine learning model.

Feature Set (FS)	Feature	Sensor Configuration (SC)	Sensor Location
FS 1	Mean	SC 1	Pelvis
	Range		Sternum (T8)
	Variance		Shoulders (left & right)
	Standard deviation		Upper legs (left & right)
	Root mean squared		Lower legs (left & right)
	Skewness	SC 2	Pelvis
	Kurtosis		Sternum (T8)
FS 2	First five FFT ^1^ coefficients		Shoulders (left & right)
FS 3	Feature set 1 and Feature set 2	SC 3	Pelvis
			Sternum (T8)

^1^ FFT, fast Fourier transformation.

**Table 3:** Data labeling criteria based on PPS ^1^ and COPV_AP ^2^ values.

Measure	Stable	Unstable
PPS	[0, 5.0)	[5.0, 10.0]
COPV_AP	[minimum, median)	[median, maximum]

^1^ PPS, perception of postural stability; ^2^ COPV_AP, mean velocity of center of pressure in anterior-posterior direction.

**Table 4:** The numbers of ACC-based measures with the abilities of factors’ effects detection.

	Effect	Main Effect			Sum	Interaction Effect				Sum
Segment		SS	LC	WP		SS × LC	SS × WP	LC × WP	SS × LC × WP	
Pelvis		27	40	43	108	2	20	40	13	75
Sternum (T8)		3	41	43	87	1	15	41	0	57
Left shoulder		4	39	43	86	1	11	43	2	57
Right shoulder		4	39	43	86	0	8	42	1	51
Left upper leg		3	41	43	87	0	12	41	9	62
Right upper leg		5	40	43	88	0	13	40	11	64
Left lower leg		19	39	43	101	17	20	39	5	81
Right lower leg		10	39	43	92	4	14	39	10	67

**Table 5:** Classification accuracies for different sensor configurations and different feature sets using different classifiers (data labeling based on COPV_AP).

	SC & FS ^1^	SC1			SC2			SC3			Average
Classifier		FS1	FS2	FS3	FS1	FS2	FS3	FS1	FS2	FS3	
KNN-City Block		90.5%	69.0%	90.7%	90.3%	70.3%	90.4%	90.6%	70.8%	90.1%	83.6%
KNN-Euclidean		90.5%	70.3%	89.8%	88.3%	69.4%	89.0%	88.7%	70.5%	88.7%	82.8%
GNB		87.8%	77.8%	88.3%	87.2%	77.5%	87.6%	87.2%	72.3%	87.5%	83.7%
KNB		86.7%	78.2%	87.0%	86.9%	79.3%	87.6%	86.7%	73.2%	86.2%	83.5%
LR		86.6%	75.8%	84.2%	89.4%	71.7%	87.9%	90.0%	71.1%	89.4%	82.9%
DA		90.3%	86.4%	90.0%	89.4%	84.1%	89.8%	90.3%	78.4%	90.1%	87.6%
SVM-Linear		89.7%	72.2%	90.6%	90.0%	71.9%	90.2%	89.9%	70.4%	89.3%	83.8%
SVM-Cubic		90.0%	82.4%	91.1%	88.3%	79.5%	89.0%	89.1%	77.8%	89.4%	86.3%
DT		87.4%	74.9%	86.0%	86.8%	71.8%	86.2%	85.3%	69.3%	85.6%	81.5%
OBT		90.3%	84.7%	90.1%	91.1%	82.1%	90.8%	89.9%	81.0%	90.3%	87.8%
OE		91.6%	86.0%	90.6%	91.5%	83.6%	91.0%	91.5%	81.8%	90.8%	88.7%
Maximum		91.6%	86.4%	91.1%	91.5%	84.1%	91.0%	91.5%	81.8%	90.8%	-
Average		89.4%	78.7%	89.1%	89.2%	77.1%	89.2%	89.2%	74.9%	89.0%	-

^1^ SC & FS, sensor configuration and feature set, the details can be found in Table 2.

**Table 6:** Classification accuracies for different sensor configurations and different feature sets using different classifiers (data labeling based on PPS).

	SC & FS ^1^	SC1			SC2			SC3			Average
Classifier		FS1	FS2	FS3	FS1	FS2	FS3	FS1	FS2	FS3	
KNN-City Block		87.4%	76.6%	86.0%	83.5%	74.9%	83.5%	85.3%	75.2%	85.6%	82.0%
KNN-Euclidean		84.2%	76.3%	85.1%	83.2%	74.8%	82.4%	84.3%	75.1%	84.0%	81.0%
GNB		83.3%	73.8%	82.0%	82.1%	73.8%	81.6%	81.3%	72.2%	80.3%	78.9%
KNB		81.3%	71.2%	80.8%	81.5%	74.5%	81.7%	81.9%	71.1%	81.2%	78.4%
LR		83.1%	77.5%	80.3%	83.8%	75.4%	82.4%	82.3%	76.0%	82.3%	80.3%
DA		84.8%	79.5%	83.8%	84.9%	78.3%	83.6%	83.2%	76.0%	83.1%	81.9%
SVM-Linear		84.7%	75.1%	84.6%	84.2%	74.8%	83.4%	83.4%	73.5%	84.2%	80.9%
SVM-Cubic		84.7%	79.7%	84.7%	82.6%	78.1%	82.2%	84.0%	75.0%	82.6%	81.5%
DT		80.2%	71.3%	78.5%	79.9%	70.4%	77.4%	80.2%	68.8%	80.9%	76.4%
OBT		84.2%	78.1%	84.5%	84.7%	78.7%	84.6%	86.1%	78.6%	85.1%	82.7%
OE		83.9%	79.3%	86.0%	85.3%	80.9%	84.4%	85.4%	78.5%	85.6%	83.3%
Maximum		87.4%	79.7%	86.0%	85.3%	80.9%	84.6%	86.1%	78.6%	85.6%	-
Average		84.1%	76.5%	83.5%	83.4%	76.3%	82.7%	83.6%	74.9%	83.4%	-

^1^ SC and FS, sensor configuration and feature set, the details of which can be found in Table 2.

**Table 7:** Classification accuracies for different feature sets using different classifiers based on Pelvis acceleration data.

	Label & FS ^1^	COPV_AP ^2^			PPS ^3^		
Classifier		FS1	FS2	FS3	FS1	FS2	FS3
KNN-City Block		89.0%	73.2%	88.5%	82.2%	75.3%	82.6%
KNN-Euclidean		88.3%	73.5%	86.3%	81.5%	75.6%	82.8%
GNB		77.9%	64.3%	76.0%	77.8%	70.0%	76.5%
KNB		85.0%	64.4%	83.4%	77.6%	68.8%	76.6%
LR		88.0%	67.9%	88.5%	80.9%	74.0%	81.9%
DA		88.7%	73.9%	88.4%	83.3%	73.9%	82.5%
SVM-Linear		88.0%	67.0%	88.4%	82.6%	73.6%	82.2%
SVM-Cubic		89.4%	73.5%	88.2%	81.5%	74.3%	79.9%
DT		85.1%	69.1%	85.5%	79.0%	69.5%	79.4%
OBT		89.2%	77.1%	89.3%	84.0%	76.0%	83.8%
OE		90.5%	79.2%	90.2%	84.1%	76.3%	84.0%
Maximum		90.5%	79.2%	90.2%	84.1%	76.3%	84.0%
Average		87.2%	71.2%	86.6%	81.3%	73.4%	81.1%

^1^ FS, feature set, the details can be found in Table 2; ^2^ COPV_AP, data set was labeled based on the COPV_AP value; ^3^ PPS, data set was labeled based on the PPS value.

## Data Availability

The date can be obtained from the corresponding author upon reasonable request.

## Appendix A

**Table A1:** Results ^1^ of *t*-tests and Analysis of variance using the acceleration data of Pelvis and T8.

ACC-Based Measures	Pelvis							Sternum (T8)						
	Main Effects			Interaction Effects				Main Effects			Interaction Effects			
	SS	LC	WP	SS × LC	SS × WP	LC × WP	SS × LC × WP	SS	LC	WP	SS × LC	SS × WP	LC × WP	SS × LC × WP
AVG_AP	*	*	*	*	+	*	*	+	*	*	+	*	*	+
AVG_ML	+	*	*	+	+	*	+	+	*	*	+	+	*	+
AVG_IS	*	*	*	+	*	*	+	+	*	*	+	+	*	+
AVG_2DR	*	*	*	+	*	*	*	+	*	*	+	*	*	+
AVG_3DR	*	*	*	+	*	*	*	+	*	*	+	+	*	+
RNG_AP	+	*	*	+	*	*	*	+	*	*	+	+	*	+
RNG_ML	+	*	*	+	*	*	+	+	*	*	+	+	*	+
RNG_IS	*	*	*	+	+	*	+	+	*	*	*	+	*	+
RNG_2DR	+	*	*	+	+	*	+	+	*	*	+	+	*	+
RNG_3DR	*	*	*	+	+	*	+	+	*	*	+	+	*	+
RMS_AP	*	*	*	*	*	*	*	+	*	*	+	*	*	+
RMS_ML	+	*	*	+	+	*	+	+	*	*	+	+	*	+
RMS_IS	*	*	*	+	+	*	+	+	*	*	+	+	*	+
RMS_2DR	*	*	*	+	+	*	+	+	*	*	+	*	*	+
RMS_3DR	*	*	*	+	+	*	+	+	*	*	+	+	*	+
ARE_SW	+	*	*	+	+	*	+	+	*	*	+	+	*	+
ARE_CC	+	*	*	+	+	*	+	+	*	*	+	+	*	+
ARE_CE	*	*	*	+	*	*	+	+	*	*	+	+	*	+
FD_CC	*	*	*	+	*	*	*	+	*	*	+	*	*	+
FD_CE	+	*	*	+	*	*	+	+	*	*	+	+	*	+
LEN_AP	+	*	*	+	*	*	+	+	*	*	+	*	*	+
LEN_ML	+	*	*	+	+	*	+	+	*	*	+	+	*	+
LEN_2DR	+	*	*	+	*	*	+	+	*	*	+	+	*	+
LEN_3DR	+	*	*	+	+	*	+	+	*	*	+	+	*	+
MD_AP	*	*	*	+	*	*	*	+	*	*	+	*	*	+
MD_ML	+	*	*	+	+	*	+	+	*	*	+	+	*	+
MD_2DR	+	*	*	+	*	*	*	+	*	*	+	*	*	+
MD_3DR	+	*	*	+	+	*	*	+	*	*	+	+	*	+
MF_AP	*	*	*	+	*	*	*	*	*	*	+	*	*	+
MF_ML	*	*	*	+	*	*	+	+	*	*	+	+	*	+
MF_2DR	*	*	*	+	*	*	*	+	*	*	+	*	*	+
MF_3DR	*	*	*	+	*	*	*	+	*	*	+	*	*	+
MV_AP	*	*	*	+	+	*	+	*	*	*	+	*	*	+
MV_ML	*	+	*	+	+	+	+	+	+	*	+	+	+	+
MV_2DR	*	+	*	+	+	+	+	+	+	*	+	+	+	+
MV_3DR	*	+	*	+	+	+	+	*	*	*	+	+	*	+
PD_P	*	*	*	+	+	*	+	+	*	*	+	+	*	+
PD_V	*	+	*	+	*	+	+	*	+	*	+	+	*	+
PP	*	*	*	+	*	*	+	*	*	*	+	+	*	+
RMSD_AP	*	*	*	+	*	*	*	+	*	*	+	*	*	+
RMSD_ML	+	*	*	+	+	*	+	+	*	*	+	+	*	+
RMSD_2DR	*	*	*	+	+	*	+	+	*	*	+	*	*	+
RMSD_3DR	*	*	*	+	+	*	+	+	*	*	+	+	*	+

^1^ Statistical significance (i.e., *p* values), * with background color: *p* < 0.05, +: *p* ≥ 0.05.

**Table A2:** Results ^1^ of *t*-tests and Analysis of variance using the acceleration data of Shoulders.

ACC-Based Measures	Left Shoulder							Right Shoulder						
	Main Effects			Interaction Effects				Main Effects			Interaction Effects			
	SS	LC	WP	SS × LC	SS × WP	LC × WP	SS × LC × WP	SS	LC	WP	SS × LC	SS × WP	LC × WP	SS × LC × WP
AVG_AP	+	*	*	+	+	*	+	+	*	*	+	+	*	+
AVG_ML	+	*	*	+	+	*	+	+	*	*	+	+	*	+
AVG_IS	+	*	*	+	*	*	+	+	*	*	+	+	*	+
AVG_2DR	+	*	*	+	+	*	+	+	*	*	+	+	*	+
AVG_3DR	+	*	*	+	+	*	+	+	*	*	+	+	*	+
RNG_AP	+	*	*	+	+	*	+	+	*	*	+	+	*	+
RNG_ML	+	*	*	+	*	*	+	+	*	*	+	+	*	+
RNG_IS	+	*	*	+	*	*	+	+	*	*	+	+	*	+
RNG_2DR	+	*	*	+	+	*	+	+	*	*	+	+	*	+
RNG_3DR	+	*	*	+	+	*	*	+	*	*	+	+	*	+
RMS_AP	+	*	*	+	+	*	+	+	*	*	+	+	*	+
RMS_ML	+	*	*	+	+	*	+	+	*	*	+	+	*	+
RMS_IS	+	*	*	+	*	*	+	+	*	*	+	+	*	+
RMS_2DR	+	*	*	+	+	*	+	+	*	*	+	+	*	+
RMS_3DR	+	*	*	+	+	*	+	+	*	*	+	+	*	+
ARE_SW	+	*	*	+	+	*	+	+	*	*	+	+	*	+
ARE_CC	+	*	*	+	+	*	+	+	*	*	+	+	*	+
ARE_CE	+	*	*	+	+	*	+	+	*	*	+	+	*	+
FD_CC	+	*	*	+	*	*	*	+	*	*	+	*	*	*
FD_CE	+	+	*	*	+	*	+	+	+	*	+	*	*	+
LEN_AP	+	*	*	+	+	*	+	+	*	*	+	+	*	+
LEN_ML	+	*	*	+	+	*	+	+	*	*	+	+	*	+
LEN_2DR	+	*	*	+	+	*	+	+	*	*	+	+	*	+
LEN_3DR	+	*	*	+	+	*	+	+	*	*	+	+	*	+
MD_AP	+	*	*	+	+	*	+	+	*	*	+	+	*	+
MD_ML	+	*	*	+	+	*	+	+	*	*	+	+	*	+
MD_2DR	+	*	*	+	+	*	+	+	*	*	+	+	*	+
MD_3DR	+	*	*	+	+	*	+	+	*	*	+	+	*	+
MF_AP	*	*	*	+	+	*	+	*	*	*	+	*	*	+
MF_ML	+	*	*	+	*	*	+	+	*	*	+	*	*	+
MF_2DR	+	*	*	+	*	*	+	+	*	*	+	*	*	+
MF_3DR	+	*	*	+	*	*	+	+	*	*	+	*	*	+
MV_AP	+	*	*	+	*	*	+	+	*	*	+	+	+	+
MV_ML	*	+	*	+	*	*	+	*	+	*	+	*	*	+
MV_2DR	*	+	*	+	*	*	+	*	+	*	+	*	*	+
MV_3DR	*	+	*	+	+	*	+	*	+	*	+	+	*	+
PD_P	+	*	*	+	+	*	+	+	*	*	+	+	*	+
PD_V	+	*	*	+	+	*	+	+	*	*	+	+	*	+
PP	+	*	*	+	+	*	+	+	*	*	+	+	*	+
RMSD_AP	+	*	*	+	+	*	+	+	*	*	+	+	*	+
RMSD_ML	+	*	*	+	+	*	+	+	*	*	+	+	*	+
RMSD_2DR	+	*	*	+	+	*	+	+	*	*	+	+	*	+
RMSD_3DR	+	*	*	+	+	*	+	+	*	*	+	+	*	+

^1^ Statistical significance (i.e., *p* values), * with background color: *p* < 0.05, +: *p* ≥ 0.05.

**Table A3:** Results ^1^ of *t*-tests and Analysis of variance using the acceleration data of Upper Legs.

ACC-Based Measures	Left Upper Leg							Right Upper Leg						
	Main Effects			Interaction Effects				Main Effects			Interaction Effects			
	SS	LC	WP	SS × LC	SS × WP	LC × WP	SS × LC × WP	SS	LC	WP	SS × LC	SS × WP	LC × WP	SS × LC × WP
AVG_AP	+	*	*	+	+	*	+	+	*	*	+	+	*	+
AVG_ML	+	*	*	+	*	*	*	*	*	*	+	*	*	*
AVG_IS	+	*	*	+	+	*	+	+	*	*	+	+	*	+
AVG_2DR	+	*	*	+	+	*	+	+	*	*	+	+	*	+
AVG_3DR	+	*	*	+	+	*	+	+	*	*	+	+	*	+
RNG_AP	+	*	*	+	+	*	+	+	*	*	+	+	*	+
RNG_ML	+	*	*	+	+	*	+	+	*	*	+	*	*	*
RNG_IS	+	*	*	+	+	*	+	+	*	*	+	+	*	+
RNG_2DR	+	*	*	+	+	*	+	+	*	*	+	+	*	+
RNG_3DR	+	*	*	+	+	*	+	+	*	*	+	+	*	+
RMS_AP	+	*	*	+	+	*	+	+	*	*	+	+	*	+
RMS_ML	+	*	*	+	*	*	*	*	*	*	+	*	*	*
RMS_IS	+	*	*	+	+	*	+	+	*	*	+	+	*	+
RMS_2DR	+	*	*	+	+	*	+	+	*	*	+	+	*	+
RMS_3DR	+	*	*	+	+	*	+	+	*	*	+	+	*	+
ARE_SW	+	*	*	+	+	*	+	+	*	*	+	+	*	+
ARE_CC	+	*	*	+	+	*	+	+	*	*	+	+	*	+
ARE_CE	+	*	*	+	+	*	+	+	*	*	+	+	*	+
FD_CC	+	*	*	+	*	*	*	+	*	*	+	*	*	*
FD_CE	+	*	*	+	*	*	+	+	*	*	+	+	*	+
LEN_AP	+	*	*	+	+	*	+	+	*	*	+	+	*	+
LEN_ML	+	*	*	+	+	*	+	+	*	*	+	*	*	*
LEN_2DR	+	*	*	+	+	*	+	+	*	*	+	+	*	+
LEN_3DR	+	*	*	+	+	*	+	+	*	*	+	+	*	+
MD_AP	+	*	*	+	+	*	+	+	*	*	+	+	*	+
MD_ML	+	*	*	+	*	*	*	+	*	*	+	*	*	*
MD_2DR	+	*	*	+	+	*	+	+	*	*	+	+	*	+
MD_3DR	+	*	*	+	+	*	+	+	*	*	+	+	*	+
MF_AP	+	*	*	+	*	*	*	+	*	*	+	*	*	*
MF_ML	*	*	*	+	*	*	*	*	*	*	+	*	*	*
MF_2DR	+	*	*	+	*	*	*	*	*	*	+	*	*	*
MF_3DR	+	*	*	+	*	*	*	+	*	*	+	*	*	*
MV_AP	+	*	*	+	*	+	+	+	+	*	+	+	+	+
MV_ML	*	*	*	+	+	*	+	*	*	*	+	+	*	+
MV_2DR	+	+	*	+	*	+	+	+	+	*	+	+	+	+
MV_3DR	*	+	*	+	+	*	+	+	+	*	+	+	+	+
PD_P	+	*	*	+	+	*	+	+	*	*	+	+	*	+
PD_V	+	*	*	+	+	*	+	+	*	*	+	*	*	+
PP	+	*	*	+	+	*	+	+	*	*	+	*	*	+
RMSD_AP	+	*	*	+	+	*	+	+	*	*	+	+	*	+
RMSD_ML	+	*	*	+	*	*	*	+	*	*	+	*	*	*
RMSD_2DR	+	*	*	+	+	*	+	+	*	*	+	+	*	+
RMSD_3DR	+	*	*	+	+	*	+	+	*	*	+	+	*	+

^1^ Statistical significance (i.e., *p* values), * with background color: *p* < 0.05, +: *p* ≥ 0.05.

**Table A4:** Results ^1^ of *t*-tests and Analysis of variance using the acceleration data of Lower legs.

ACC-Based Measures	Left Lower Leg							Right Lower Leg						
	Main Effects			Interaction Effects				Main Effects			Interaction Effects			
	SS	LC	WP	SS × LC	SS × WP	LC × WP	SS × LC × WP	SS	LC	WP	SS × LC	SS × WP	LC × WP	SS × LC × WP
AVG_AP	+	*	*	*	*	*	+	+	*	*	+	+	*	+
AVG_ML	+	*	*	*	*	*	+	+	*	*	+	+	*	+
AVG_IS	*	*	*	+	+	*	+	*	*	*	*	+	*	+
AVG_2DR	+	*	*	*	*	*	+	+	*	*	+	+	*	+
AVG_3DR	*	*	*	*	+	*	+	+	*	*	*	+	*	+
RNG_AP	+	*	*	+	*	*	+	+	*	*	+	+	*	+
RNG_ML	+	*	*	+	+	*	+	+	*	*	+	+	*	*
RNG_IS	*	*	*	+	+	*	+	*	*	*	+	+	*	+
RNG_2DR	+	*	*	+	+	*	+	+	*	*	+	+	*	+
RNG_3DR	+	*	*	+	+	*	+	+	*	*	+	+	*	+
RMS_AP	+	*	*	*	*	*	+	+	*	*	+	+	*	+
RMS_ML	+	*	*	*	*	*	+	+	*	*	+	+	*	+
RMS_IS	*	*	*	+	+	*	+	*	*	*	+	+	*	+
RMS_2DR	+	*	*	*	*	*	+	+	*	*	+	+	*	+
RMS_3DR	*	*	*	*	+	*	+	+	*	*	*	+	*	+
ARE_SW	+	*	*	+	+	*	+	+	*	*	+	+	*	+
ARE_CC	+	*	*	+	+	*	+	+	*	*	+	+	*	+
ARE_CE	+	*	*	*	+	*	+	+	*	*	+	+	*	+
FD_CC	*	*	*	*	*	*	*	+	*	*	+	*	*	*
FD_CE	*	*	*	+	+	*	+	+	*	*	*	+	*	+
LEN_AP	*	*	*	+	*	*	+	+	*	*	+	*	*	+
LEN_ML	+	*	*	+	+	*	+	+	*	*	+	*	*	+
LEN_2DR	*	*	*	+	*	*	+	+	*	*	+	*	*	+
LEN_3DR	*	*	*	+	+	*	+	*	*	*	+	+	*	+
MD_AP	+	*	*	*	*	*	+	+	*	*	+	+	*	+
MD_ML	+	*	*	+	+	*	+	+	*	*	+	+	*	*
MD_2DR	+	*	*	+	*	*	+	+	*	*	+	+	*	*
MD_3DR	*	*	*	+	+	*	+	*	*	*	+	+	*	+
MF_AP	*	*	*	*	*	*	*	*	*	*	+	*	*	*
MF_ML	*	*	*	+	*	*	*	*	*	*	+	*	*	*
MF_2DR	*	*	*	*	*	*	*	*	*	*	+	*	*	*
MF_3DR	*	*	*	*	*	*	*	*	*	*	+	*	*	*
MV_AP	*	+	*	+	+	+	+	+	+	*	+	*	+	+
MV_ML	*	+	*	+	+	+	+	+	+	*	+	*	+	+
MV_2DR	*	+	*	+	+	+	+	+	+	*	+	*	+	+
MV_3DR	+	+	*	+	+	+	+	+	+	*	+	*	+	+
PD_P	+	*	*	*	*	*	+	+	*	*	+	+	*	+
PD_V	+	*	*	+	*	*	+	+	*	*	+	*	*	*
PP	+	*	*	+	*	*	+	+	*	*	+	*	*	*
RMSD_AP	+	*	*	*	*	*	+	+	*	*	+	+	*	+
RMSD_ML	+	*	*	+	+	*	+	+	*	*	+	+	*	+
RMSD_2DR	+	*	*	+	+	*	+	+	*	*	+	+	*	+
RMSD_3DR	*	*	*	*	+	*	+	*	*	*	+	+	*	+

^1^ Statistical significance (i.e., *p* values), * with background color: *p* < 0.05, +: *p* ≥ 0.05.

## References

[1] Huang C.W., Der Sue P., Abbod M.F., Jiang B.C., Shieh J.S. (2013). Measuring center of pressure signals to quantify human balance using multivariate multiscale entropy by designing a force platform. Sensors.

[2] Duarte M., Freitas S.M.S.F. (2010). Revision of posturography based on force plate for balance evaluation. Rev. Bras. Fisioter..

[3] Hsiao H., Simeonov P. (2001). Preventing falls from roofs: A critical review. Ergonomics.

[4] Son S.M. (2016). Influence of Obesity on Postural Stability in Young Adults. Osong Public Health Res. Perspect..

[5] Jebelli H., Ahn C.R., Stentz T.L. (2016). Fall risk analysis of construction workers using inertial measurement units: Validating the usefulness of the postural stability metrics in construction. Saf. Sci..

[6] Lord S.R., Sherrington C., Menz H.B. (2001). Falls in older people: Epidemiology, risk factors and strategies for prevention. Age Ageing.

[7] Chi C.F., Chang T.C., Ting H.I. (2005). Accident patterns and prevention measures for fatal occupational falls in the construction industry. Appl. Ergon..

[8] Passmore D., Chae C., Borkovskaya V., Baker R., Yim J.H. (2019). Severity of U.S. Construction Worker Injuries, 2015–2017. E3S Web Conf..

[9] Schneider S.P. (2001). Musculoskeletal Injuries in Construction: A Review of the Literature. Appl. Occup. Environ. Hyg..

[10] Gauchard G., Chau N., Mur J.M., Perrin P. (2001). Falls and working individuals: Role of extrinsic and intrinsic factors. Ergonomics.

[11] Guo L., Kou J., Xiong S. (2020). Subjective and Objective Measures to Assess Postural Instability: Their Linear Correlations and Abilities to Detect Effects of Work-Related Factors. Proceedings of the International Conference on Applied Human Factors and Ergonomics.

[12] DiDomenico A., Nussbaum M.A. (2005). Interactive effects of mental and postural demands on subjective assessment of mental workload and postural stability. Saf. Sci..

[13] Guo L., Xiong S. (2020). Effects of working posture, lifting load, and standing surface on postural instability during simulated lifting tasks in construction. Ergonomics.

[14] Schieppati M., Tacchini E., Nardone A., Tarantola J., Corna S. (1999). Subjective perception of body sway. J. Neurol. Neurosurg. Psychiatry.

[15] Tversky A., Kahneman D. (1978). Judgment under Uncertainty: Heuristics and Biases. Uncertain. Econ..

[16] Lin D., Seol H., Nussbaum M.A., Madigan M.L. (2008). Reliability of COP-based postural sway measures and age-related differences. Gait Posture.

[17] Qiu H., Xiong S. (2015). Center-of-pressure based postural sway measures: Reliability and ability to distinguish between age, fear of falling and fall history. Int. J. Ind. Ergon..

[18] Horak F.B., Shupert C.L., Mirka A. (1989). Components of postural dyscontrol in the elderly: A review. Neurobiol. Aging.

[19] Maki B.E., Holliday P.J., Fernie G.R. (1990). Aging and Postural Control. J. Am. Geriatr. Soc..

[20] Prieto T.E., Myklebust J.B., Hoffmann R.G., Lovett E.G., Myklebust B.M. (1996). Measures of postural steadiness: Differences between healthy young and elderly adults. IEEE Trans. Biomed. Eng..

[21] Clark R.A., Bryant A.L., Pua Y., McCrory P., Bennell K., Hunt M. (2010). Validity and reliability of the Nintendo Wii Balance Board for assessment of standing balance. Gait Posture.

[22] Karatsidis A., Bellusci G., Schepers H., de Zee M., Andersen M., Veltink P. (2016). Estimation of Ground Reaction Forces and Moments During Gait Using Only Inertial Motion Capture. Sensors.

[23] Faber G.S., Chang C.C., Kingma I., Dennerlein J.T., van Dieën J.H. (2016). Estimating 3D L5/S1 moments and ground reaction forces during trunk bending using a full-body ambulatory inertial motion capture system. J. Biomech..

[24] Hamacher D., Bertram D., Fölsch C., Schega L. (2012). Evaluation of a visual feedback system in gait retraining: A pilot study. Gait Posture.

[25] Cloete T., Scheffer C. Repeatability of an off-the-shelf, full body inertial motion capture system during clinical gait analysis. Proceedings of the 2010 Annual International Conference of the IEEE Engineering in Medicine and Biology.

[26] Shippen J., May B. (2016). Constitutive kinematic modes and shapes during vehicle ingress/egress. Appl. Ergon..

[27] Kim S., Nussbaum M.A. (2013). Performance evaluation of a wearable inertial motion capture system for capturing physical exposures during manual material handling tasks. Ergonomics.

[28] Baydal-Bertomeu J.M., Durá-Gil J.V., Piérola-Orcero A., Parrilla Bernabé E., Ballester A., Alemany-Munt S. (2016). A PCA-based bio-motion generator to synthesize new patterns of human running. PeerJ Comput. Sci..

[29] Carson H.J., Collins D., Richards J. (2016). Initiating technical refinements in high-level golfers: Evidence for contradictory procedures. Eur. J. Sport Sci..

[30] de Magalhaes F.A., Vannozzi G., Gatta G., Fantozzi S. (2015). Wearable inertial sensors in swimming motion analysis: A systematic review. J. Sports Sci..

[31] Carson H.J., Collins D., Richards J. (2014). Intra-individual movement variability during skill transitions: A useful marker?. Eur. J. Sport Sci..

[32] Eckardt F., Münz A., Witte K. (2014). Application of a Full Body Inertial Measurement System in Dressage Riding. J. Equine Vet. Sci..

[33] Starrs P., Chohan A., Fewtrell D., Richards J., Selfe J. (2012). Biomechanical differences between experienced and inexperienced wheelchair users during sport. Prosthet. Orthot. Int..

[34] Supej M. (2010). 3D measurements of alpine skiing with an inertial sensor motion capture suit and GNSS RTK system. J. Sports Sci..

[35] Doric I., Frison A.-K., Wintersberger P., Riener A., Wittmann S., Zimmermann M., Brandmeier T. (2016). A Novel Approach for Researching Crossing Behavior and Risk Acceptance. Proceedings of the 8th International Conference on Automotive User Interfaces and Interactive Vehicular Applications Adjunct—Automotive’UI 16.

[36] Ruch W.F., Platt T., Hofmann J., Niewiadomski R., Urbain J., Mancini M., Dupont S. (2014). Gelotophobia and the Challenges of Implementing Laughter into Virtual Agents Interactions. Front. Hum. Neurosci..

[37] Damian I., Kistler F., Obaid M., Buhling R., Billinghurst M., Andre E. Motion capturing empowered interaction with a virtual agent in an Augmented Reality environment. Proceedings of the 2013 IEEE International Symposium on Mixed and Augmented Reality (ISMAR).

[38] Giggins O.M., Sweeney K.T., Caulfield B. (2014). Rehabilitation exercise assessment using inertial sensors: A cross-sectional analytical study. J. Neuroeng. Rehabil..

[39] Neville C., Ludlow C., Rieger B. (2015). Measuring postural stability with an inertial sensor: Validity and sensitivity. Med. Devices Evid. Res..

[40] Grimm B., Bolink S. (2016). Evaluating physical function and activity in the elderly patient using wearable motion sensors. EFORT Open Rev..

[41] Liu J., Zhang X., Lockhart T.E. (2012). Fall risk assessments based on postural and dynamic stability using inertial measurement unit. Saf. Health Work.

[42] Handelzalts S., Alexander N.B., Mastruserio N., Nyquist L.V., Strasburg D.M., Ojeda L.V. (2020). Detection of Real-World Trips in At-Fall Risk Community Dwelling Older Adults Using Wearable Sensors. Front. Med..

[43] Kutílek P., Socha V., Čakrt O., Svoboda Z. (2015). Assessment of postural stability in patients with cerebellar disease using gyroscope data. J. Bodyw. Mov. Ther..

[44] Abe Y., Sugaya T., Sakamoto M. (2014). Postural control characteristics during single leg standing of individuals with a history of ankle sprain: Measurements obtained using a gravicorder and head and foot accelerometry. J. Phys. Ther. Sci..

[45] Rouis A., Rezzoug N., Gorce P. (2014). Validity of a low-cost wearable device for body sway parameter evaluation. Comput. Methods Biomech. Biomed. Engin..

[46] Cruz-Montecinos C., De la Fuente C., Rivera-Lillo G., Morales-Castillo S., Soto-Arellano V., Querol F., Pérez-Alenda S. (2017). Sensory strategies of postural sway during quiet stance in patients with haemophilic arthropathy. Haemophilia.

[47] Moe-Nilssen R. (1998). Test-retest reliability of trunk accelerometry during standing and walking. Arch. Phys. Med. Rehabil..

[48] Craig J.J., Bruetsch A.P., Lynch S.G., Horak F.B., Huisinga J.M. (2017). Instrumented balance and walking assessments in persons with multiple sclerosis show strong test-retest reliability. J. Neuroeng. Rehabil..

[49] Mayagoitia R.E., Lötters J.C., Veltink P.H., Hermens H. (2002). Standing balance evaluation using a triaxial accelerometer. Gait Posture.

[50] Grewal G.S., Schwenk M., Lee-Eng J., Parvaneh S., Bharara M., Menzies R.A., Talal T.K., Armstrong D.G., Najafi B. (2015). Sensor-Based Interactive Balance Training with Visual Joint Movement Feedback for Improving Postural Stability in Diabetics with Peripheral Neuropathy: A Randomized Controlled Trial. Gerontology.

[51] Greene B.R., McGrath D., Walsh L., Doheny E.P., McKeown D., Garattini C., Cunningham C., Crosby L., Caulfield B., Kenny R.A. (2012). Quantitative falls risk estimation through multi-sensor assessment of standing balance. Physiol. Meas..

[52] Moe-Nilssen R., Helbostad J.L. (2002). Trunk accelerometry as a measure of balance control during quiet standing. Gait Posture.

[53] Gago M.F., Fernandes V., Ferreira J., Silva H., Rodrigues M.L., Rocha L., Bicho E., Sousa N. (2015). The effect of levodopa on postural stability evaluated by wearable inertial measurement units for idiopathic and vascular Parkinson’s disease. Gait Posture.

[54] Mancini M., Salarian A., Carlson-Kuhta P., Zampieri C., King L., Chiari L., Horak F.B. (2012). ISway: A sensitive, valid and reliable measure of postural control. J. Neuroeng. Rehabil..

[55] Park J.-H., Mancini M., Carlson-Kuhta P., Nutt J.G., Horak F.B. (2016). Quantifying effects of age on balance and gait with inertial sensors in community-dwelling healthy adults. Exp. Gerontol..

[56] Frames C., Soangra R., Lockhart T.E. (2013). Assessment of postural stability using inertial measurement unit on inclined surfaces in healthy adults. Biomed. Sci. Instrum..

[57] Bzdúšková D., Valkovič P., Hirjaková Z., Kimijanová J., Hlavačka F. (2018). Parkinson’s disease versus ageing: Different postural responses to soleus muscle vibration. Gait Posture.

[58] Ehsani H., Mohler J., Marlinski V., Rashedi E., Toosizadeh N. (2018). The influence of mechanical vibration on local and central balance control. J. Biomech..

[59] Hasegawa N., Shah V.V., Carlson-Kuhta P., Nutt J.G., Horak F.B., Mancini M. (2019). How to Select Balance Measures Sensitive to Parkinson’s Disease from Body-Worn Inertial Sensors—Separating the Trees from the Forest. Sensors.

[60] Baracks J., Casa D.J., Covassin T., Sacko R., Scarneo S.E., Schnyer D., Yeargin S.W., Neville C. (2018). Acute sport-related concussion screening for collegiate athletes using an instrumented balance assessment. J. Athl. Train..

[61] Ghislieri M., Gastaldi L., Pastorelli S., Tadano S., Agostini V. (2019). Wearable Inertial Sensors to Assess Standing Balance: A Systematic Review. Sensors.

[62] Gordt K., Gerhardy T., Najafi B., Schwenk M. (2018). Effects of Wearable Sensor-Based Balance and Gait Training on Balance, Gait, and Functional Performance in Healthy and Patient Populations: A Systematic Review and Meta-Analysis of Randomized Controlled Trials. Gerontology.

[63] Hubble R.P., Naughton G.A., Silburn P.A., Cole M.H. (2015). Wearable Sensor Use for Assessing Standing Balance and Walking Stability in People with Parkinson’s Disease: A Systematic Review. PLoS ONE.

[64] Chiou S., Bhattacharya A., Lai C.-F., Succop P.A. (1998). Effect of environmental and task risk factors on workers ’ perceived sense of postural sway and instability. Occup. Ergon..

[65] Min S.-N., Kim J.-Y., Parnianpour M. (2012). The effects of safety handrails and the heights of scaffolds on the subjective and objective evaluation of postural stability and cardiovascular stress in novice and expert construction workers. Appl. Ergon..

[66] Chiu Y.L., Tsai Y.J., Lin C.H., Hou Y.R., Sung W.H. (2017). Evaluation of a smartphone-based assessment system in subjects with chronic ankle instability. Comput. Methods Programs Biomed..

[67] Raymakers J.A., Samson M.M., Verhaar H.J.J. (2005). The assessment of body sway and the choice of the stability parameter(s). Gait Posture.

[68] Martinez-Mendez R., Sekine M., Tamura T. (2012). Postural sway parameters using a triaxial accelerometer: Comparing elderly and young healthy adults. Comput. Methods Biomech. Biomed. Engin..

[69] Xsens-Tutorials: Preparing hardware MVN Link. https://tutorial.xsens.com/video/preparing-hardware-mvn-link.

[70] Maxwell S.E., Delaney H.D. (2003). Designing Experiments and Analyzing Data: A Model Comparison Perspective, Second Edition.

[71] MathWorks Help Center—Classification Learner App: Choose Classifier Options. https://www.mathworks.com/help/stats/choose-a-classifier.html#bunt0rb-1.

[72] Flach P.A. (2003). The Geometry of ROC Space: Understanding Machine Learning Metrics through ROC Isometrics. Proceedings, Twent. Int. Conf. Mach. Learn..

[73] Fawcett T. (2006). An introduction to ROC analysis. Pattern Recognit. Lett..

[74] Liu Y., Redmond S.J., Wang N., Blumenkron F., Narayanan M.R., Lovell N.H. (2011). Spectral analysis of accelerometry signals from a directed-routine for falls-risk estimation. IEEE Trans. Biomed. Eng..

[75] Narayanan M.R., Redmond S.J., Scalzi M.E., Lord S.R., Celler B.G., Lovell N.H. (2010). Longitudinal falls-risk estimation using triaxial accelerometry. IEEE Trans. Biomed. Eng..

[76] Saunders N.W., Koutakis P., Kloos A.D., Kegelmeyer D.A., Dicke J.D., Devor S.T. (2015). Reliability and validity of a wireless accelerometer for the assessment of postural sway. J. Appl. Biomech..

[77] Whitney S.L., Roche J.L., Marchetti G.F., Lin C.C., Steed D.P., Furman G.R., Musolino M.C., Redfern M.S. (2011). A comparison of accelerometry and center of pressure measures during computerized dynamic posturography: A measure of balance. Gait Posture.

[78] BROWN H.J., SIEGMUND G.P., GUSKIEWICZ K.M., VAN DEN DOEL K., CRETU E., BLOUIN J.-S. (2014). Development and Validation of an Objective Balance Error Scoring System. Med. Sci. Sport. Exerc..

[79] Guo L., Xiong S. (2017). Accuracy of Base of Support Using an Inertial Sensor Based Motion Capture System. Sensors.

[80] Toosizadeh N., Mohler J., Armstrong D.G., Talal T.K., Najafi B. (2015). The influence of diabetic peripheral neuropathy on local postural muscle and central sensory feedback balance control. PLoS ONE.

[81] Bonora G., Mancini M., Carpinella I., Chiari L., Ferrarin M., Nutt J.G., Horak F.B. (2017). Investigation of anticipatory postural adjustments during One-Leg Stance using inertial sensors: Evidence from subjects with Parkinsonism. Front. Neurol..

[82] Fransson P.A., Gomez S., Patel M., Johansson L. (2007). Changes in multi-segmented body movements and EMG activity while standing on firm and foam support surfaces. Eur. J. Appl. Physiol..

[83] Honegger F., Hubertus J.W., Allum J.H.J. (2013). Coordination of the head with respect to the trunk, pelvis, and lower leg during quiet stance after vestibular loss. Neuroscience.

[84] Quijoux F., Vienne-Jumeau A., Bertin-Hugault F., Zawieja P., Lefèvre M., Vidal P.-P., Ricard D. (2020). Center of pressure displacement characteristics differentiate fall risk in older people: A systematic review with meta-analysis. Ageing Res. Rev..

[85] Huurnink A., Fransz D.P., Kingma I., van Dieën J.H. (2013). Comparison of a laboratory grade force platform with a Nintendo Wii Balance Board on measurement of postural control in single-leg stance balance tasks. J. Biomech..

[86] Chang W.-D., Chang W.-Y., Lee C.-L., Feng C.-Y. (2013). Validity and Reliability of Wii Fit Balance Board for the Assessment of Balance of Healthy Young Adults and the Elderly. J. Phys. Ther. Sci..

[87] Clark R.A., McGough R., Paterson K. (2011). Reliability of an inexpensive and portable dynamic weight bearing asymmetry assessment system incorporating dual Nintendo Wii Balance Boards. Gait Posture.

[88] Lajoie Y., Gallagher S.P. (2004). Predicting falls within the elderly community: Comparison of postural sway, reaction time, the Berg balance scale and the Activities-specific Balance Confidence (ABC) scale for comparing fallers and non-fallers. Arch. Gerontol. Geriatr..

[89] Salonen L., Kivelä S.L. (2012). Eye diseases and impaired vision as possible risk factors for recurrent falls in the aged: A systematic review. Curr. Gerontol. Geriatr. Res..

[90] Sturnieks D.L., George R.S., Lord S.R. (2008). Balance disorders in the elderly. Neurophysiol. Clin..

[91] Muir S.W., Gopaul K., Montero Odasso M.M. (2012). The role of cognitive impairment in fall risk among older adults: A systematic review and meta-analysis. Age Ageing.

[92] Deandrea S., Lucenteforte E., Bravi F., Foschi R., La Vecchia C., Negri E. (2010). Risk factors for falls in community-dwelling older people: A systematic review and meta-analysis. Epidemiology.

[93] Jebelli H., Ahn C.R., Stentz T.L. (2016). Comprehensive Fall-Risk Assessment of Construction Workers Using Inertial Measurement Units: Validation of the Gait-Stability Metric to Assess the Fall Risk of Iron Workers. J. Comput. Civ. Eng..

[94] Simeonov P.I., Hsiao H., Dotson B.W., Ammons D.E. (2003). Control and perception of balance at elevated and sloped surfaces. Hum. Factors.

[95] Ambrose A.F., Paul G., Hausdorff J.M. (2013). Risk factors for falls among older adults: A review of the literature. Maturitas.

[96] DiDomenico A., McGorry R.W., Huang Y.-H., Blair M.F. (2010). Perceptions of postural stability after transitioning to standing among construction workers. Saf. Sci..

[97] DiDomenico A., McGorry R.W., Banks J.J. (2011). Effects of common working postures on balance control during the stabilisation phase of transitioning to standing. Ergonomics.

